# Neglected bilateral triphalangeal of the thumb delta type in adult case: A case report

**DOI:** 10.1016/j.ijscr.2023.109087

**Published:** 2023-11-24

**Authors:** Meirizal, Hilmi Muhammad, Brahmantyo Danang Guntoro, Rahadyan Magetsari

**Affiliations:** Dept. of Orthopedic and Traumatology Sardjito General Hospital, Faculty of Medicine, Public Health and Nursing, Universitas Gadjah Mada, Yogyakarta, Indonesia

**Keywords:** Case report, Congenital deformity, Triphalangeal thumb, Thumb deformity, Hand reconstruction, Triphalangeal thumb management

## Abstract

**Introduction:**

In rare case, thumb has extra phalanges known as triphalangeal of the thumb (TPT). Patients with TPT can have difficulty doing work/activities that require high precision. Therefore, surgical intervention is essential. This report provides an approach for a patient with TPT.

**Presentation of case:**

A patient with TPT who underwent removal of extra phalanges and arthrodesis of interphalangeal (IP) joints is presented. The left thumb deviated 25^o^ to ulnar while the contralateral part deviated 15^o^ to radial. X-ray revealed both thumbs had extra delta-shaped middle phalanges. Complete excision of extra phalanges and simple arthrodesis of IP joints with two K-wires in 10° to 15° flexion was performed. Healing process ended without any complications and the patient had an improvement.

**Discussion:**

Productive-age patients with TPT can have difficulty doing work and activities that require high precision, especially in the non-opposable type of the right hand. Furthermore, the female patient is highly emphasizing the cosmetics of her hand to increase her self-confidence. Therefore, surgical intervention is essential for this patient. We performed complete excision of extra phalanges and simple arthrodesis of IP joints with two K-wires in 10° to 20° flexion. The first K-wire is introduced intramedullary as a primary fixator for longitudinal alignment, and the second wire is inserted obliquely as an anti-rotation wire. Functional outcome was assessed after 6 months post-removal of the wire which gave a satisfying result.

**Conclusion:**

TPT is a rare anomaly which surgical intervention can improve the appearance and the precision of the hand.

## Highlights

• TPT in adults is a rare condition since most TPT patients have already undergone a correction surgery in early life.
• Surgical intervention is essential to give an acceptable appearance and also to improve the precision of the hand.
• In adult TPT patients, removal of accessory bone followed by arthrodesis is another modality that offers a favorable outcome.

## 1 Introduction

The first finger of the palm, the thumb, generally only has two bones inside of it. However, on rare occasions, some humans have more than two bones. In 1559, the first case of three bones of the thumb was described by Columbo et al. (1559). Later, this condition is known as triphalangeal of the thumb (TPT). The epidemiology of TPT in Southeast Asia is unknown since the condition is rare. In the Netherlands, it is known that TPT occurs in people with a 1:16,000 ratio [1]. Nowadays, it turns out that TPT is predominantly an inheritable condition in a dominant manner [2]. If the TPT is inherited, both thumbs are affected.

Based on opposition movement, TPT can be classified as opposable or non-opposable [3]. Rarely, TPT can be accompanied by a syndrome such as Holt-Oram syndrome or Laurin-Sandrow syndrome [1,4]. When TPT is followed by these syndromes, the thumb is usually hypoplastic and cannot do an opposition movement. The opposition movement is maintained in isolated TPT, though its power is reduced. Sometimes, TPT can be accompanied by polydactyly. Based on the shape of the extra phalanx, TPT can be divided into delta, trapezoid, and complete types. Furthermore, the presentation of syndactyly can classify the TPT into Triphalangeal Thumb-Polysyndactyly Syndrome (TPT-PS), Haas-type Polysyndactyly, and Laurin-Sandrow Syndrome (LSS), as proposed by experts [4].

Clinically, patients with TPT have a ridiculously long thumb, accompanied by deviation of the thumb due to the length and shape of the extra phalanx [1,5]. It can be thumb-like or more finger-like. Depending on the length of the thumb and the presence of hypoplastic muscle, TPT can be hypermobile at the metacarpophalangeal (MP) joint, while the carpometacarpal (CMC) joint can be hypoplastic, malformed, or absent [1]. Due to this condition, the CMC joint becomes less mobile and diminished, particularly in the finger-like thumb. In the early years, TPT patients may not complain of any disturbance caused by TPT. As time goes on, patients may encounter difficulties in daily activities that require oppositional movement and precise movement, such as writing, picking up a small object, etc. On other occasions, the patient may not experience these difficulties but feel unconfident with the appearance of their thumb. Generally, parents are the ones who seek a correctional surgical procedure.

Since its findings, however, there has been no guideline regarding how to approach a patient with TPT of any age. Hence, this report provides an approach for a patient with TPT. Also, the work has been reported in line with the SCARE criteria [6].

## 2 Case report

A 23-year-old female presented to our hospital with bilateral congenital malformed thumbs. The patient had understood her condition since she was a child and refused to undergo surgical correction. In adulthood, the patient encountered two main problems. First, she usually felt unconfident due to the cosmetic appearance of her thumbs. Second, those thumbs disturbed her activities when precise movements were needed, such as sewing. This activity, sewing, is essential for her job as a tailor. As time went on, the patient felt more uncomfortable with her thumbs since they made sewing difficult. Finally, the patient and her parents sought medical advice for her thumbs.

**Fig. 1:**
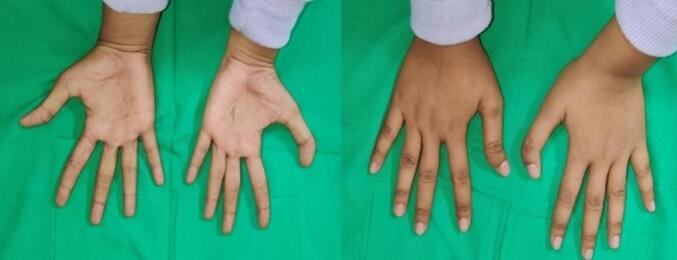
Clinical picture of both thumbs at pre-operative condition.

Physical examination showed that the left thumb deviated 25° to the ulnar side, while the contralateral thumb deviated 15° to the radial side without any swelling in either thumb (Fig. 1). There was no sign of pain or inflammation in either thumb. Both thumbs also did not suffer from limited movement in abduction and flexion. Other than the malformation, there was no medical condition such as anemia or a history of trauma. The Disability of Arm, Shoulder, and Hand (DASH) score was evaluated to assess the specific disability of the extremity, and the result was 17.5. The Sollerman Hand Function (SHF) and Michigan Hand Outcome were also evaluated, giving scores of 56 and 60 for the left and right hand on the SHF test, and 65.6 and 66.6 for the left and right hand on the Michigan Hand Outcome.

**Fig. 2:**
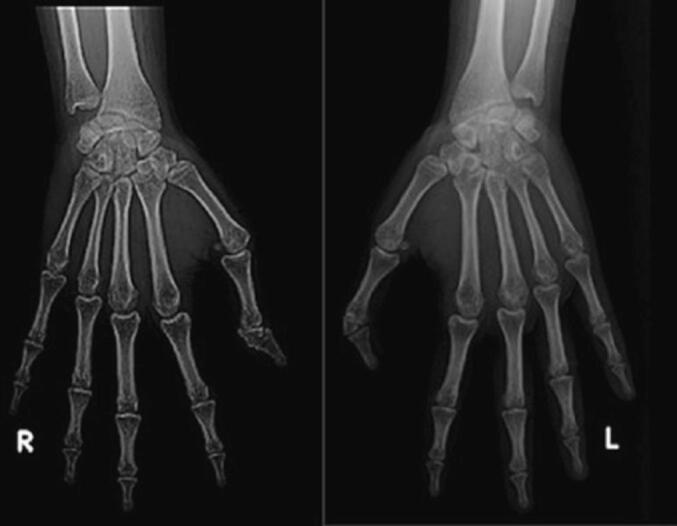
X-ray of both thumbs at pre-operative condition.

Under the plain x-ray of the thumb (Fig. 2), it was revealed that both thumbs had an extra delta-shaped bone between two normal bones at the distal interphalangeal (DIP) joint. Hence, the patient was diagnosed with bilateral delta-type TPT.

The patient underwent operation for the correction of both thumbs. In general anesthesia, an H-shaped incision was made on the dorsal side. To expose the accessory phalanx, the extensor tendon was split longitudinally from the PIP joint through the DIP joint. Then, the accessory phalanx was removed, followed by ligament and tendon reconstruction.

**Fig. 3:**
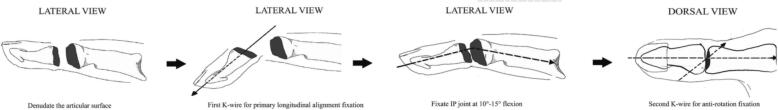
Representative operative procedure of the left hand with TPT [7].

The distal phalanx and the proximal phalanx were placed close together with denudation of the articular surface. The first K-wire was introduced retrogradely to the distal phalanx intramedullary with the joint flexed to gain an optimal view, then with an antegrade fashion, advanced the K-wire to the proximal phalanx. After the IP joint was in adequate contact, the IP joint was bent in a 10°–15° flexion position. The first K-wire was the primary fixator for longitudinal alignment. Then, the second K-wire was introduced obliquely over the IP joint. The second K-wire acted as an antirotation wire to stabilize the joint (Fig. 3). After 3 days of observation, there were no complications.

**Fig. 4:**
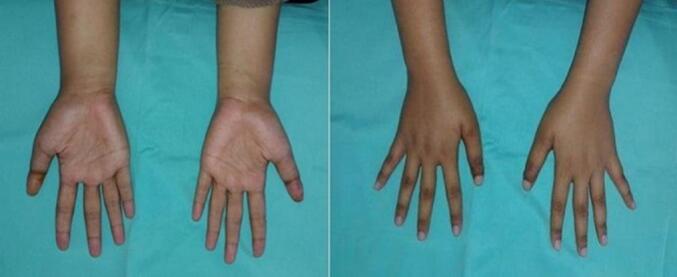
Clinical picture of both thumbs at six months post-operative condition.

Six months later, during the follow-up, the K-wire was removed (Fig. 4). There were no chief complaints or adverse events before or after the K-wire removal. To examine the function of the hand after the correctional surgery, the SHF test was executed. The patient also performed tasks with her hands, such as inserting a thread into a pinhole precisely, and returned to work earlier. Both of her hands had a satisfying outcome, with an overall score of 80 and 79 in the dominant and non-dominant hands, respectively. The Michigan Hand Outcome score was 97.2 and 98.9, and the DASH Score was 7.

## 3 Discussion

**Fig. 5:**
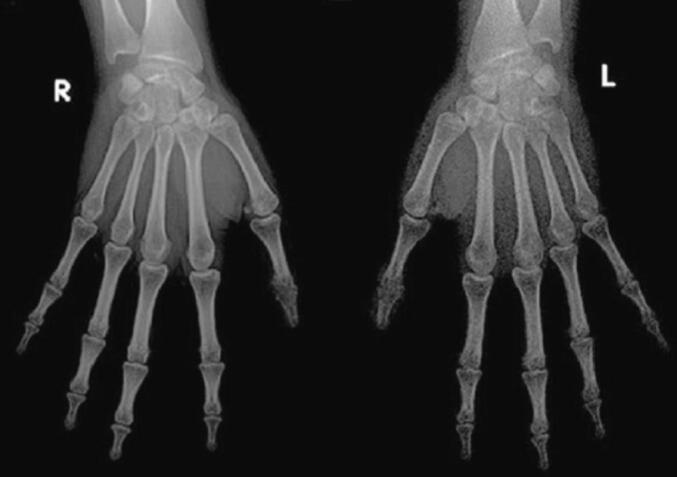
X-ray of both thumbs at six months post-operative condition.

TPT is a rare congenital anomaly with an incidence of 1 in 25,000 live births. It is not common to find TPT cases in young adults because, usually, parents of TPT children seek correction surgery early in life. An adult with TPT may find difficulties whenever they need to do precise work. Most surgical intervention has been done in TPT with other conditions such as polydactyly, syndactyly, web contracture, and thenar muscle atrophy [4]. In contrast, this patient does not have any other medical condition other than TPT. Regardless, surgical intervention is essential for this patient to achieve a more precise movement and regain her confidence by improving the appearance of patient's thumbs (Fig. 5).

Since 1976, TPT correction surgery has consisted of 3 interventions: removal of the accessory phalanx, arthrodesis of the interphalangeal joint, and reduction osteotomies [8]. The removal of the accessory phalanx corrects the angulation and length of the thumb; however, this procedure may reduce the stability of the thumb. Furthermore, the removal of additional phalanxes requires a reconstruction of the collateral ligament. Moreover, according to a report [9], the power of the thumb with TPT diminished significantly when compared with the unaffected thumb, although reconstruction surgery had been done. They assumed the surgical intervention might spare the anatomical correction without any significant improvement in the thumb's function. In some experts study [10], most TPT patients had hypoplastic thenar, and this is the sole cause of diminished function of the thumb. Therefore, it is recommended for TPT patients under 2 years old to allow early cerebral integration of the reconstructed thumb. In the case of teenage or adult TPT, the stability of the joint can be partially achieved with the help of K-wire insertion.

In this case report, a simple accessory phalanx removal and arthrodesis were performed without reductive osteotomies. To achieve the desired stability of the joint, two non-parallel K-wires were inserted for six months. The first K-wire has a role as a primary fixator for longitudinal alignment, and the second wire functions as an anti-rotation wire. In comparison with Woods' procedure that has been mentioned before, this approach is much simpler, and the outcome is favorable for the patient, as proven by the SHF and DASH assessments, where both give a good result.

Nowadays, an exploration of genetic mutation has become popular, especially in congenital anomalies. Some genetic mutations have been described in the literature [2,11]. It may give a better understanding of the pathology of TPT itself and help further research to prevent the development of congenital TPT. In this report, genetic testing is not done because the findings of genetic mutations do not affect the treatment.

TPT in adults is a rare condition since most TPT patients have already undergone a correction surgery in early life as per their parents' consent. Nevertheless, it is not impossible to find TPT cases in older patients. Surgical intervention is essential to improve appearance and the precision of the hand to perform a high precision task. In adult TPT patients, removal of accessory bone followed by arthrodesis is another modality that offers a favorable outcome.

## 4 Conclusion

TPT in adults is a rare condition since most TPT patients have already undergone a correction surgery in early life as per their parents' consent. Nevertheless, it is not impossible to find TPT cases in older patients. Surgical intervention is essential not only to give an acceptable appearance but also to improve the precision of the hand to perform a task that requires high precision. In adult TPT patients, removal of accessory bone followed by arthrodesis is another modality that offers a favorable outcome.

## Declaration of informed consent

Written consent for the publication of this case report and its accompanying images has been acquired from the patient (or their guardian or next of kin). There is no information in submitted manuscript that can be used to identify the patient.

## Ethical approval

Ethical clearance is not required for this case report, according to our institution's research ethics committee. The committee has verified that the report adheres to standard clinical practices and does not involve experimental interventions or the need for additional data collection.

## Funding

The author(s) received NO financial support for the preparation, research, authorship, and/or publication of this manuscript.

## Author contribution

Conceptualization: M.M., H.M., B.D.G., R.M.

Data Curation: M.M., H.M., B.D.G., R.M.

Formal Analysis: M.M., H.M., B.D.G., R.M.

Funding Acquisition: Not applicable.

Investigation: M.M., H.M., B.D.G.

Project Administration: M.M., H.M., B.D.G.

Resources: M.M., H.M., B.D.G., R.M.

Software: Not applicable.

Supervision: M.M., H.M., R.M.

Validation: M.M., H.M., B.D.G., R.M.

Visualization: M.M., H.M., B.D.G., R.M.

Writing-original draft preparation: M.M., H.M., B.D.G., R.M.

Writing-review and editing: M.M., H.M., B.D.G., R.M.

## Guarantor

Meirizal.

## Research registration number

This case report is not a first-in-man case.

## Conflict of interest statement

The author(s) do NOT have any potential conflicts of interest with respect to this manuscript.
